# In vivo rectal dosimeter with MRI marker

**DOI:** 10.1371/journal.pone.0354149

**Published:** 2026-07-22

**Authors:** Euntaek Yoon, Jin Dong Cho, Chang Heon Choi, Jin Ho Kim, Jeong Ho Kim, Jong Min Park, Jung-in Kim

**Affiliations:** 1 Interdisciplinary program in Bioengineering, Graduate School, Seoul National University, Seoul, Republic of Korea; 2 Biomedical Research Institute, Seoul National University College of Medicine, Seoul, Republic of Korea; 3 Department of Radiation Oncology, Seoul National University Hospital, Seoul, Republic of Korea; 4 Institute of Radiation Medicine, Seoul National University Medical Research Center, Seoul, Republic of Korea; 5 Department of Radiation Oncology, Kangbuk Samsung Hospital, Sungkyunkwan University School of Medicine, Seoul, Republic of Korea; 6 Department of Radiation Oncology, Seoul National University College of Medicine, Seoul, Republic of Korea; 7 Department of Radiation Oncology, Samsung Changwon Hospital, Sungkyunkwan University School of Medicine, Changwon, Republic of Korea; Indiana University School of Medicine, UNITED STATES OF AMERICA

## Abstract

**Background:**

Hypofractionated external beam radiotherapy for prostate cancer necessitates precise rectal dose evaluation. We fabricated a radiochromic polyurethane-based in vivo rectal dosimeter with a custom MRI marker and a patient-specific applicator for in vivo dose verification (IDV) during gated MR-image guided radiotherapy (MR-IGRT).

**Methods:**

The dosimeter featured a radiochromic polyurethane active layer, incorporating leucomalachite green (LMG) and tartrazine. To accommodate anatomical variations, a detachable PMMA applicator was designed in five sizes. For localization, four elastomeric materials, including two polyurethane-based and two silicone-based materials, were evaluated as candidate MRI markers. Post-irradiation fading was assessed over time to evaluate measurement stability. A dose-response calibration was performed to establish a linear relationship between net optical density (OD) and absorbed dose. Furthermore, dose uncertainty was analyzed based on the law of error propagation. For verification, in vivo measurements were conducted for two patients and compared with TPS-calculated doses.

**Results:**

Vyta Flex 20 was selected as the optimal MRI marker due to its high signal intensity and ease of fabrication. Post-irradiation net OD showed a dose-dependent temporal response, and the readout time was standardized to 2 h. The dosimeter’s sensitivity was 0.00253 cGy^-1^. Dose uncertainties were determined to be 2.1%, 2.0%, and 1.6% at 100, 200, and 300 cGy, respectively. In vivo verification showed mean dose differences of 3.7 ± 1.4% (95% CI, 0.1–7.3%) for patient #1 and 5.9 ± 2.1% (95% CI, 0.8–11.1%) for patient #2. Measured doses were consistently higher than TPS-calculated doses, suggesting possible contributions from localization uncertainty in high-dose-gradient regions and the material-dependent response of the radiochromic active layer.

**Conclusion:**

The fabricated radiochromic dosimeter with a custom MRI marker and adjustable applicator demonstrated preliminary feasibility as a proof-of-concept system for in vivo rectal dose verification during MR-IGRT. Further studies with larger patient cohorts and more treatment fractions are required to validate its reproducibility, statistical robustness, and clinical utility. Further refinement in positioning is also needed, particularly in dose-gradient regions.

## 1. Introduction

Previous clinical studies have demonstrated that external beam radiation therapy (EBRT) is effective for treating prostate cancer [1]. To compensate for both interfractional and intrafractional motion during EBRT for prostate cancer, planning target volume (PTV) margins of 3–10 mm are typically used, depending on the image-guidance technique employed [2,3]. However, large target margins—particularly those exceeding 5–7 mm posteriorly—can significantly increase the overlap with nearby organs at risk such as the rectum, leading to higher rectal doses and potential toxicity, including rectal bleeding [4]. Therefore, the dose delivered to the rectum is the main limiting factor in increasing the prescribed doses of EBRT for prostate cancer. As hypofractionated radiotherapy is increasingly employed in clinics [5,6], the evaluation and management of the dose delivered to the rectum have become more important.

To reduce the interfractional target margins, adaptive radiation therapy (ART) can be used [7]; however, ART cannot be easily performed in clinics owing to its complicated procedure, high labor intensity, and the cumulative imaging dose of up to about 0.16 Gy in 5-fraction SBRT or up to about 1.3 Gy in conventional 40-fraction RT when ART is performed with daily kilo-voltage cone beam computed tomography (CBCT) [8]. To reduce the intrafractional target margins of EBRT for prostate cancer, magnetic resonance image-guided radiation therapy (MR-IGRT) has been increasingly used because it provides excellent soft-tissue contrast without delivering additional imaging dose to the patient [9]. MR-IGRT can monitor the locations of the prostate and rectum during treatment and perform gated radiotherapy based on their positions [10,11]. Although gated MR-IGRT may help ensure delivery of the prescribed dose to the target volume, it does not directly verify the dose delivered to the rectum during treatment.

The rigorous quality assurance (QA) method for beams delivered to the rectum involves in vivo dose verification (IDV) [12]. The IDV method for the rectum involves attaching a dosimeter to endorectal equipment such as a transrectal ultrasound (TRUS) probe and a rectal balloon. [12–16] The use of an endorectal balloon can reduce intrafraction prostate motion and may allow smaller intrafraction margins [17]. In addition, plastic scintillation detectors mounted on an endorectal balloon enable real-time point-dose measurements near the rectal wall. However, measurements using TRUS probes and MOSFET have significant positional uncertainty [12,16].

In this study, an in vivo rectal dosimeter was fabricated using a radiochromic polymer. Additionally, for the localization of the dosimeter, a bead-shaped magnetic resonance imaging (MRI) marker was produced and used for verification.

## 2. Materials and methods

### 2.1. Design and fabrication of the dosimeter

**Fig 1:**
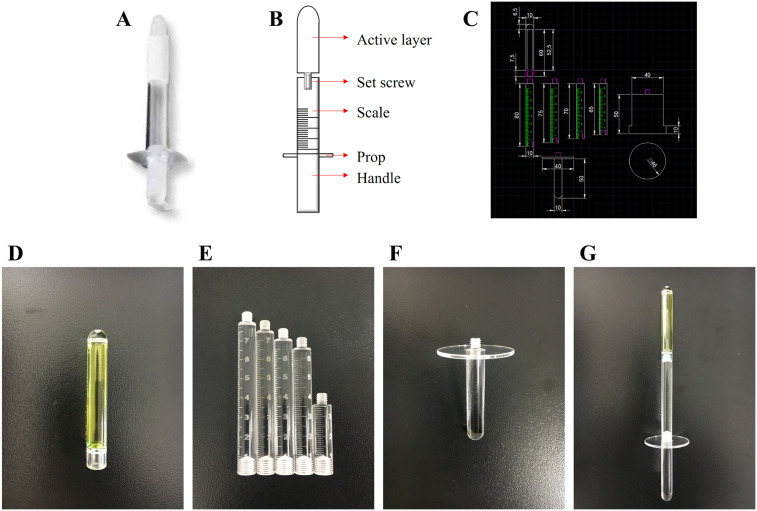
(a) Commercial anoscope tubes (HEINE UniSpec^®^ Disposable Tubes), (b) drawing of a rectal dosimeter modeled after an anoscope tube, (c) design drawing of a patient-specific rectal dosimeter, (d) radiochromic polyurethane in a hemispherical cylinder container, (e) detachable and length-adjustable body, (f) detachable handle part, (g) rectal dosimeter.

The in vivo rectal dosimeter comprises an active layer, a sealable container, and an applicator equipped with a scale to determine the insertion length into the body. The overall design followed the shape of a commercial anoscope tube (HEINE UniSpec® Disposable Tubes, HEINE Optotechnik GmbH & Co. KG, Herrsching, Germany), as shown in Fig 1A and 1B. To enable patient-specific in vivo use, we designed a detachable dosimeter body (Fig 1C) in five sizes (10 × 151.5, 10 × 181.5, 10 × 186.5, 10 × 191.5, and 10 × 196.5 mm) to accommodate patient-to-patient differences in rectal length. The radiochromic active layer was housed within the container and connected to the patient-specific applicator (Fig 1D and 1E), and the assembly was completed by attaching it to the handle (Fig 1F and 1G). The handle, sealable container, and applicator were all fabricated from polymethyl methacrylate (PMMA).

The dosimeter’s active layer consisted of radiochromic polyurethane, a polyurethane-based polymer incorporating an initiator and a radiochromic dye. The names and weight percentages of the materials used are listed in Table 1. The polyurethane consisted of Crystal Clear® Series and Clear Flex™ Series (Smooth-On, Inc., Macungie, USA), which consisted of Parts A and B. Part A contained diisocyanate, whereas Part B contained polyol. In Part B, the initiator tetrabromomethane and the radiochromic dye leucomalachite green (LMG) were added, and the organic solvents dimethyl sulfoxide and acetone were introduced for dissolution. The solution was incubated for 30 min at room temperature. Finally, after adding Part A to Part B of the solution, the completed radiochromic polyurethane was injected into the container. To remove air bubbles generated during injection, the container was placed in a pressure chamber and 60 psi was applied for 48 h.

**Table 1:** Material composition of radiochromic active layer.

Material name	Weight percent (%)
Polyurethane	90.95%
Tetrabromomethane	4.00%
Leucomalachite green	2.00%
Tartrazine	0.05%
Dimethyl sulfoxide	1.00%
Acetone	2.00%

The container, including the active layer and other components, was sterilized for clinical use using ethylene oxide. The sterilized rectal dosimeter was stored in a space not exposed to light. Figs 1C–1G show the drawings, components, and final product of the rectal dosimeter. The finalized rectal dosimeter met the biocompatibility acceptance criteria of ISO 10993−5 and ISO 10993−10, showing no evidence of cytotoxicity (cell viability: 96.82%–99.04%) or rectal irritation in the rabbit model [18,19].

### 2.2. Evaluation of candidate materials for MRI markers

In MR images, it is difficult to distinguish between radiochromic polyurethane, acrylic, and tissue; therefore, it is difficult to specify the location of the rectal dosimeter in MR images captured by the ViewRay MRIdian (ViewRay, Inc., Oakwood Village, OH, USA). Such localization errors can lead to significant discrepancies between the doses calculated by the treatment planning system (TPS) and the actual measured doses.

While commercial MRI markers like PinPoint® are available, their liquid state poses a risk of mixing with the active layer, which may alter the dosimeter's properties. Furthermore, our internal testing showed that PinPoint® solidified with gelatin failed to produce detectable MR signals at small dimensions. To address these issues, we developed custom-fabricated MRI markers. Although a CuSO₄-based marker was initially produced following the method in [20], it was found to undergo an undesirable chemical reaction with the active layer. Therefore, we fabricated MRI-marker specimens using four elastomeric candidate materials—two polyurethane-based materials (PMC 780 and Vyta Flex 20) and two silicone-based materials (Dragon Skin and Body Double)—and evaluated their MR signal intensities.

### 2.3. Scanning protocol

A radiochromic polymer-based dosimeter measures absorbed dose, which is derived from the change in optical density (OD) between pre- and post-irradiation images using a calibration curve. In this study, OD value was obtained through scanning with a cone-beam Optical CT Scanner, Vista™ Optical CT Scanner (Modus Medical Devices Inc., Ontario, Canada). A diffuse light source and a bandpass filter with a central wavelength of 633 nm were used. The scan was rotated by 360°, and 512 images were acquired. The acquired images were reconstructed in 3D using the Vista 3-D Reconstruction software (Modus Medical Devices Inc., Ontario, Canada). The settings used for image acquisition and reconstruction are presented in Table 2.

**Table 2:** Image acquisition and reconstruction setting.

Acquisition			Reconstruction	
Shutter speed (ms)	Frame rate (fps)	Resolution (pixel)	Dimensions	Voxel size (mm^3^)
25.0	7.5	640 × 480	256 × 256 × 256	0.5 × 0.5 × 0.5

OD values were obtained from the reconstructed images using MicroView™ software (Parallax Innovations, Ontario, Canada). Because absolute OD can be affected by baseline non-uniformity and background/offset signals in the optical CT system, the response was evaluated using background-corrected net OD. Net OD values for each radiation dose were calculated using Equation (1):


$$\begin{array}{c} \text{net OD}={\text{log}}_{\text{10}}\frac{I_{unirr}-I_{bgd}}{I_{irr}-I_{bgd}} \end{array}$$ (1)

where $I_{irr}$ is the intensity of the light passing through the irradiated active layer, $I_{unirr}$ is the intensity of the light passing through the active layer, and $I_{bgd}$ is the background intensity measured in the absence of the active layer. To reduce light-scattering artifacts, the aquarium was filled with silicone oil (KF-54; Shin-Etsu Chemical Co., Ltd., Tokyo, Japan).

### 2.4. Dosimetric characteristics of rectal dosimeter

#### 2.4.1. Post-irradiation fading assessment.

To establish stable measurement conditions, post-irradiation fading was evaluated as a function of time, with measurements performed at 30, 60, 120, 240, and 360 min post-irradiation. The radiochromic polyurethane in cuvettes was irradiated with 6 MV photon beams to doses of 50, 100, and 300 cGy using a Varian TrueBeam linear accelerator (Varian Medical Systems Inc., Palo Alto, California, USA). The intended dose was delivered using a lateral beam arrangement, and three cuvette samples were prepared for each dose level. Net OD was measured over time after irradiation. For analysis, an 8 × 8 × 8 mm³ region of interest (ROI) was defined at the center of the cuvette. The mean OD across all voxels within the ROI was used as the representative OD for each time point, and net OD was then calculated from these representative OD values.

#### 2.4.2. Dose-response calibration.

The sensitivity of the rectal dosimeter was evaluated by irradiating the dosimeter with 6 MV photon beams to absorbed doses of 0, 20, 50, 80, 100, 200, and 300 cGy. During optical CT scanning, a cylindrical ROI (6 mm in diameter and 9 mm in height) centered on the active layer region was used for readout. Sensitivity was defined as the slope of the linear fit of net OD as a function of absorbed dose. For each dose point, the mean OD across all voxels within the ROI was used as the representative OD, and net OD was calculated from these mean OD values. The voxel-wise OD standard deviation within the ROI was also obtained and propagated to estimate the uncertainty in net OD.

#### 2.4.3. Dose uncertainty analysis.

The dose uncertainty of the rectal dosimeter was determined using the law of error propagation. Following the methodology described in [21,22], the relative uncertainty in the absorbed dose was calculated as follows:


$$\begin{array}{c} \frac{\sigma_{D}}{D}=\;\sqrt{{\left(\frac{x\sigma_{\alpha }}{D}\right)}^{\text{2}}+{\left(\frac{\alpha \sigma_{x}}{D}\right)}^{\text{2}}} \end{array}$$ (2)

where D is the absorbed dose, x is the net OD, and α is the slope of the linear calibration curve. $\sigma_{x}$ and $\sigma_{\alpha }$ represent the standard uncertainties in the net OD measurement and the fitted slope, respectively. This approach accounts for both the measurement variability and the uncertainty derived from the fitting parameters of the calibration curve.

### 2.5. In vivo measurement

Patients were prospectively recruited between 21 December 2018 and 20 December 2019 at Seoul National University Hospital. The study protocol was approved by the Institutional Review Board of Seoul National University Hospital (IRB No. 1811-139-989). Written informed consent was obtained from all participants prior to enrollment. No minors were included.

In vivo dosimetry was conducted using a rectal dosimeter with an MRI marker, and the measured dose was compared with the dose calculated using the TPS. For each measured fraction, the daily MR image acquired before treatment was used as the reference image for dose comparison. The planning CT and MR images were deformably registered to the daily MR image in the MRIdian TPS. The dosimeter ROI was defined on the daily MR image using the MRI-visible marker and the known geometry of the active layer. To maintain consistency across fractions, the same ROI size and shape were used for all fraction-specific comparisons. The dose distribution was recalculated on the deformed daily anatomy, and the mean TPS-calculated dose within the dosimeter ROI was extracted. This mean dose was then compared with the corresponding optical-CT-based measured dose. The comparison was performed separately for each measured fraction, and dose accumulation across fractions was not performed. In vivo measurements were conducted for two patients with prostate cancer, each treated with a 5-fraction scenario-gated MR-IGRT and a fraction dose of 7.25 Gy. Three fractions were treated with a plan involving a rectal dosimeter, whereas the remaining two fractions were treated without the rectal dosimeter. Consequently, the TPS and measured doses were compared thrice per patient.

Because the in vivo evaluation included only two patients and three measured fractions per patient, no formal hypothesis testing was performed. For each patient, the percentage dose differences between the TPS-calculated and measured doses were reported as the mean ± sample standard deviation, together with exploratory 95% confidence intervals calculated using the t-distribution.

## 3. Results

### 3.1. MRI marker material selection and fabrication

**Fig 2:**
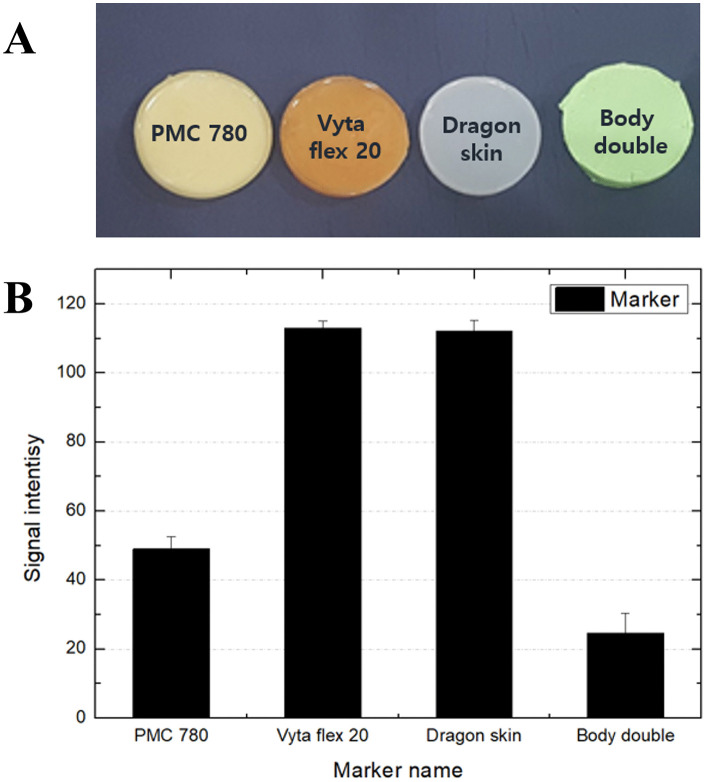
(A) MRI-marker specimens fabricated from four elastomeric candidate materials. (B) Signal intensity of each marker expressed as mean ± standard deviation. The signal intensities were 49.00 ± 3.57 (PMC 780), 113.00 ± 2.19 (Vyta Flex 20), 112.00 ± 3.22 (Dragon Skin), and 25.60 ± 5.85 (Body Double).

As shown in Fig 2B, the signal intensity (mean ± standard deviation) was 49.00 ± 3.57 for PMC 780, 113.00 ± 2.19 for Vyta Flex 20, 112.00 ± 3.22 for Dragon Skin, and 25.60 ± 5.85 for Body Double. Notably, the signal intensities of Vyta Flex 20 and Dragon Skin were more than two times higher than that of PMC 780, and more than four times higher than that of Body Double. Based on these experimental results, Vyta Flex 20, a polyurethane-based material in the same material class as Crystal Clear 200 used in the rectal dosimeter, was selected as the MRI-marker material because of its ease of fabrication and reduced bubble formation without vacuum degassing, unlike Dragon Skin. The MRI-marker bead was made in the form of a cylinder with a diameter of 6 mm and a height of 3 mm.

### 3.2. Dosimetric characteristics of rectal dosimeter

#### 3.2.1. Determination of the post-irradiation readout time.

**Fig 3:**
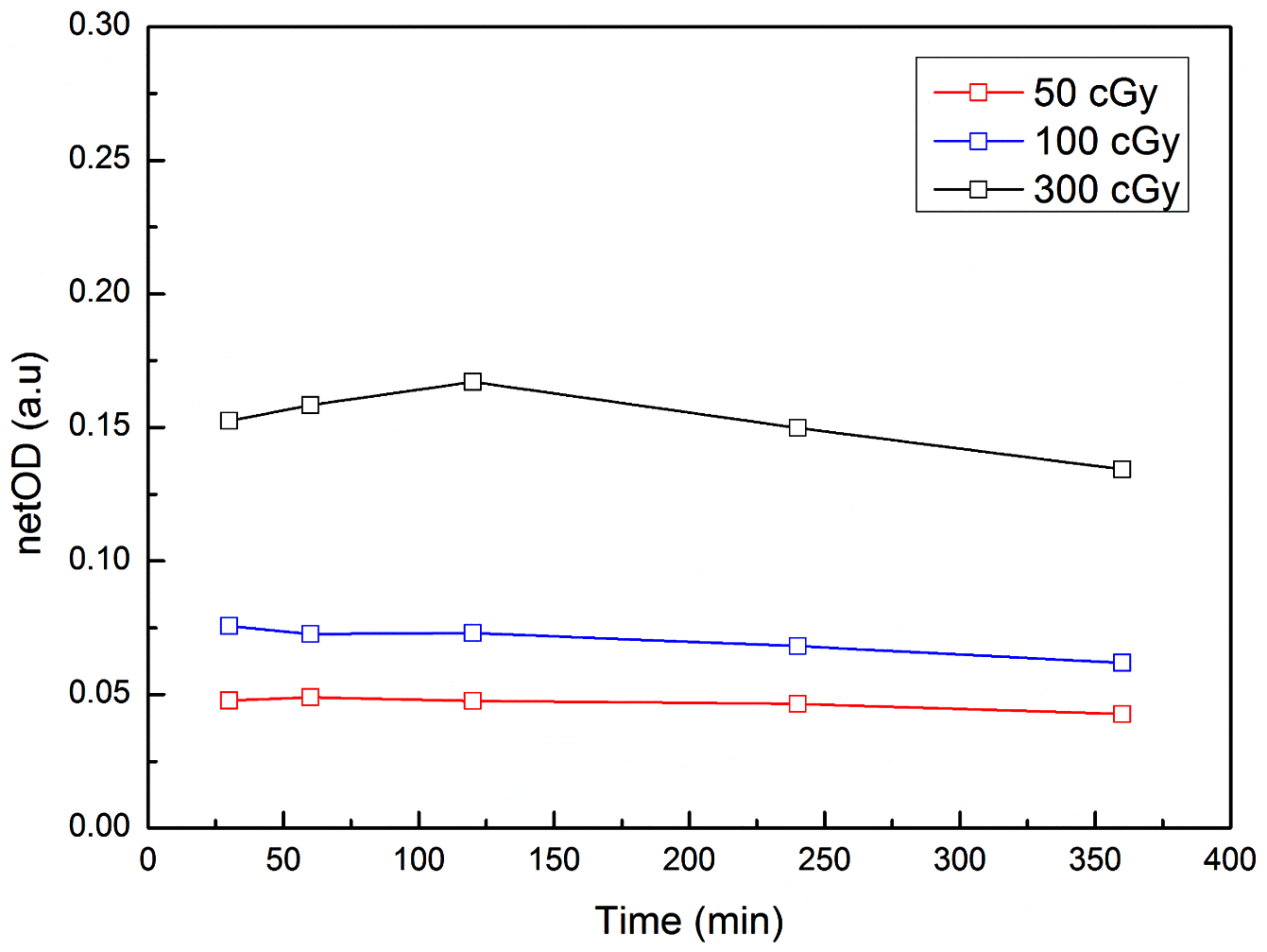
Net OD changes over time for each dose.

The change in net OD of the rectal dosimeter was evaluated as a function of post-irradiation scan time. As shown in Fig 3, net OD at 300 cGy slightly increased up to 120 min and then decreased, whereas the variations at 50 and 100 cGy were minimal over the same period. To standardize the readout and minimize time-dependent variability, all rectal dosimetry measurements were acquired 2 h post-irradiation. A slight net OD increase was observed at 300 cGy up to 120 min, likely due to measurable post-irradiation coloration at high dose. At 50 and 100 cGy, the magnitude of this effect is small relative to readout variability, and thus no clear increase is observed.

#### 3.2.2. Dose-response calibration curve.

**Fig 4:**
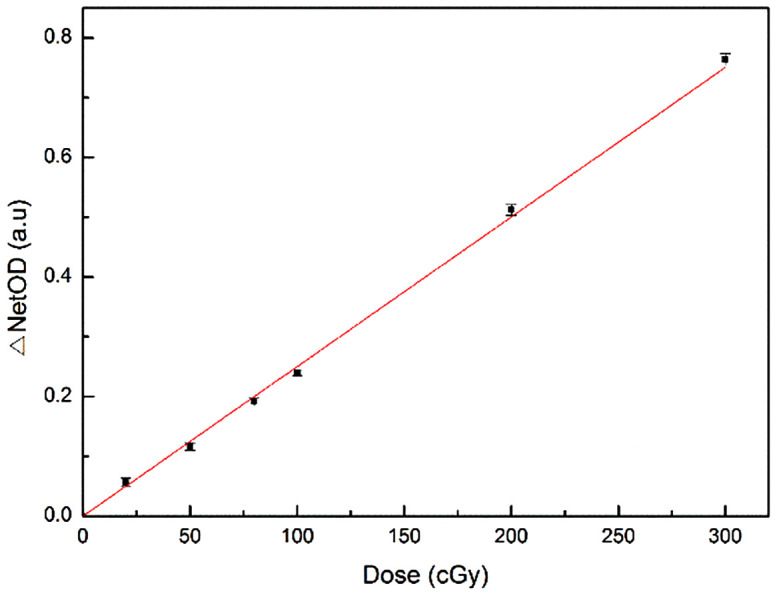
Net optical density as a function of absorbed dose.

The sensitivity according to the absorbed dose when the rectal dosimeter was irradiated with 6 MV photons is shown in Fig 4. Through linear calibration, sensitivity was evaluated as 0.00253 cGy^-1^, and R^2^ was determined to be 0.9994.

#### 3.2.3. Dose uncertainty.

**Fig 5:**
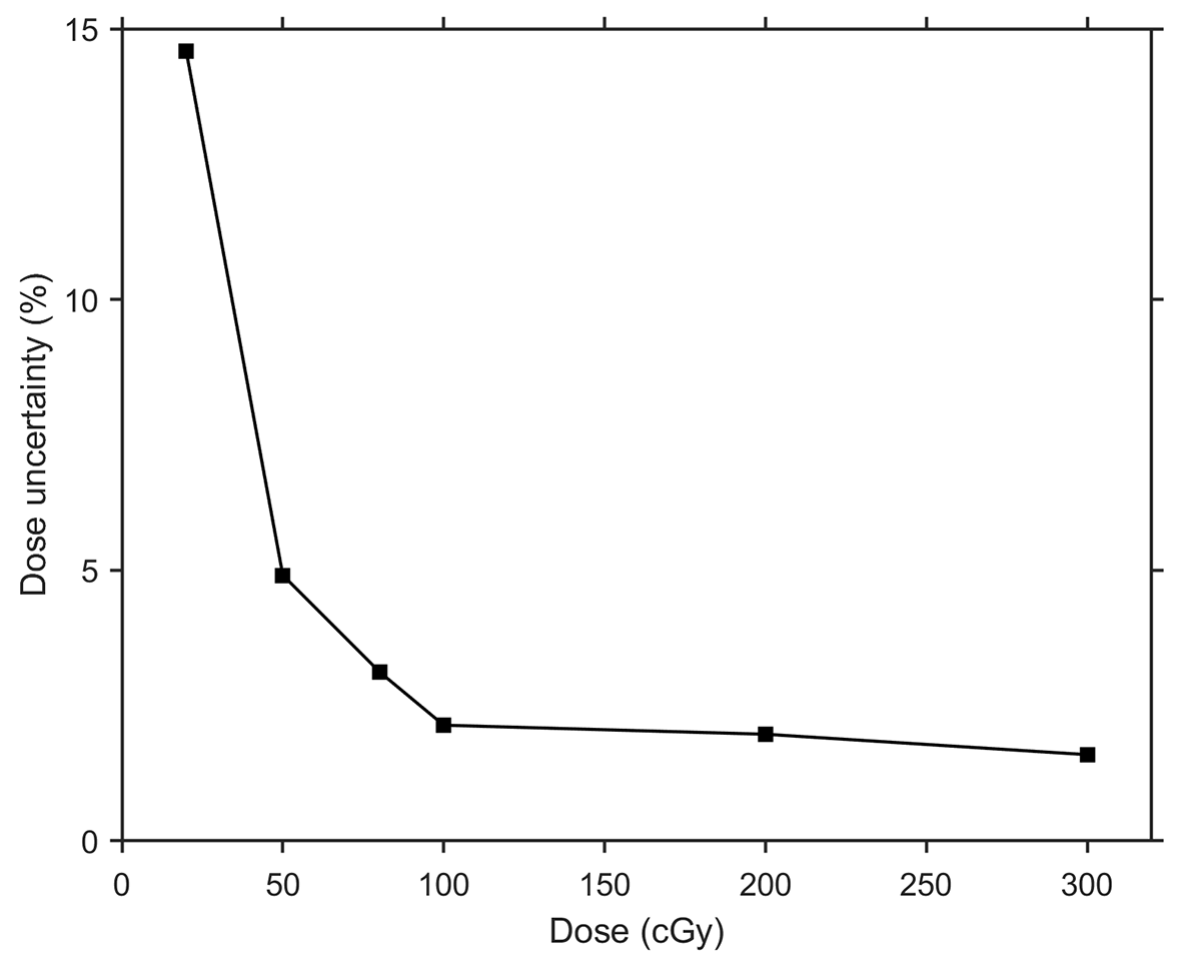
Dose uncertainty of the rectal dosimeter as a function of the absorbed dose.

The dose uncertainties of the rectal dosimeter were 2.1%, 2.0% and 1.6% at 100, 200, and 300 cGy, respectively. As shown in Fig 5, dose uncertainty increases below 100 cGy because σₓ in Eq. (2) becomes relatively large in the low-dose region.

### 3.3. In vivo measurement results

**Fig 6:**
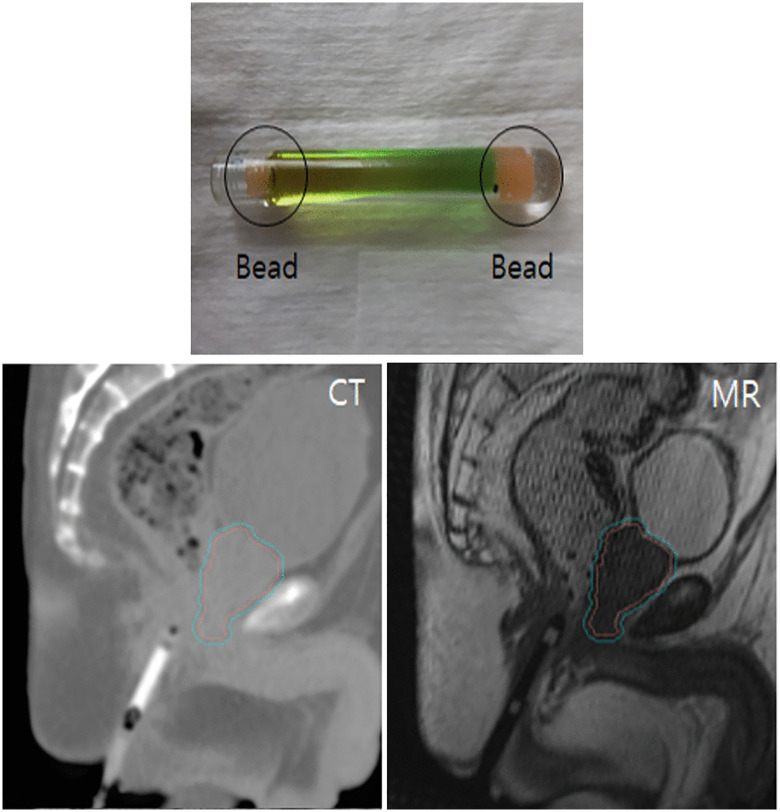
Final product of rectal in vivo dosimeter with MRI marker (upper row) and CT and MR image when the rectal dosimeter was applied to the patient (lower row).

Fig 6 shows the rectal in vivo dosimeter with an MRI marker applied in the clinical study, in addition to the CT and MR images when the dosimeter was located within the patient.

Finally, the dose values measured through in vivo rectal dosimetry were compared with the dose values obtained through the TPS calculations, as summarized in Table 3.

**Table 3:** Comparison of dose measured from Day 1 to Day 3 with dose calculated from treatment planning system.

Patient #1	Calculated dose (cGy)	Measured dose (cGy)	Difference (%)
Day 1	343.3	362.8	5.4
Day 2	342.0	352.3	2.9
Day 3	340.7	350.9	2.9
Patient #2	Calculated dose (cGy)	Measured dose (cGy)	Difference (%)
Day 1	107.0	114.2	6.3
Day 2	118.3	128.3	7.8
Day 3	287.0	297.9	3.7

The mean difference and standard deviation between the TPS-calculated and measured doses over the three fractions were 3.7 ± 1.4% for patient #1 and 5.9 ± 2.1% for patient #2. The corresponding 95% confidence intervals were 0.1–7.3% and 0.8–11.1%, respectively (Table 3).

## 4. Discussion

In this study, we fabricated an in vivo rectal dosimeter based on a radiochromic polyurethane active layer. Additionally, we custom-fabricated a polyurethane-based MRI marker that did not react with the active layer for use in MR-IGRT. In this proof-of-concept clinical study, we compared the measured and TPS-calculated doses to evaluate the preliminary feasibility of the rectal dosimeter for IDV.

The designed rectal dosimeter allows for length adjustment tailored to the specific rectal anatomy of the patient, which is crucial for maintaining positional reproducibility (see Fig 1). This feature addresses a common challenge observed in other in vivo rectal dosimeters [12,16], where a device shorter than the rectum may shift, compromising detector localization. While MR deformation tracking assists in monitoring the probe's position, further investigation is required to refine accurate localization and fixation techniques. Furthermore, the fabricated dosimeter successfully passed biocompatibility, cytotoxicity, and rectal irritation tests, justifying its clinical safety. Although the Vyta Flex 20 MRI marker was not explicitly included in these tests, no additional testing was deemed necessary as it is a solid polyurethane-based material—consistent with the radiochromic material used—and does not leak within the assembly.

The active layer utilized a polyurethane-based material containing LMG. Tartrazine, a food-grade coloring agent, was added to enhance sensitivity without altering the inherent radiochromic properties, a method validated in previous literature [23]. In MR imaging, distinguishing between the radiochromic active layer, PMMA, and human tissue is challenging, necessitating the use of markers for accurate identification. The markers in this study were required to be solid-state and non-reactive with the active layer while providing sufficient signal intensity for MR visualization. Following comparative testing of the four elastomeric candidate materials shown in Fig 2, Vyta Flex 20 was selected as the optimal MRI-marker material.

Fig 3 illustrates the change in net optical density (OD) as a function of post-irradiation time. The readout time was standardized to 2 h post-irradiation to standardize the readout during the dose-dependent post-irradiation temporal response While the net OD remained stable at 50 and 100 cGy, a slight increase followed by a decrease was observed at 300 cGy. This phenomenon is likely attributed to post-irradiation coloration kinetics: at higher doses, a greater concentration of radiation-induced reactive species may continue to oxidize the radiochromic components shortly after irradiation, leading to a measurable increase in OD. Conversely, at lower doses, these changes remain within the range of measurement variability. Although the 2 h readout time was selected to ensure measurement stability, it may limit applications requiring immediate dose feedback; therefore, the present system is more suitable for post-treatment in vivo dose verification than for real-time dose monitoring. Earlier readouts may be possible using dose- and time-dependent correction models, but a universal correction factor was not applied because the post-irradiation response varied with dose, particularly at 300 cGy. Future studies with additional time points and dose levels are needed to establish such correction models.

Figs 4 and 5 illustrate the calibration curves derived from the net OD values of the active layer material as a function of the absorbed dose, along with an analysis of the corresponding dose uncertainty. The dose uncertainty was calculated to be < 3% for absorbed doses exceeding 1 Gy; this information should be taken into account when interpreting dosimetric results. To contextualize the dosimetric characteristics of the proposed system, Table 4 provides a brief comparison of representative in vivo dosimetry tools for rectal dose verification, including reported dosimetric performance, spatial information, real-time capability, and MRI compatibility. The proposed system does not provide immediate dose feedback, unlike MOSFET/MOSkin and plastic scintillation detectors, but may fill a gap as an MRI-localizable, post-treatment rectal dosimetry system with potential for three-dimensional dose assessment during MR-IGRT.

**Table 4:** Comparison of representative in vivo dosimetry systems for rectal dose verification.

System	Reported dosimetric performance*	Spatial information	Real-time capability	MRI compatibility
MOSFET/MOSkin detectors	Phantom agreement: within ±5%; total uncertainty: 5.7% [14]	Point dose	Yes	Limited
Plastic scintillation detectors	Phantom agreement: 0.5%; clinical measured–planned difference: −0.4 ± 2.8% [16]	Point dose	Yes	Possible
Gel / polymer dosimeters	Measured–planned dose difference: 1.6–1.8%; DTA: 0.61–0.63 mm [24]	3D dose	No	Possible
Proposed dosimeter (this study)	Dose uncertainty: 2.1%, 2.0%, and 1.6% at 100, 200, and 300 cGy	3D possible	No	Yes

* Reported values are representative literature-reported metrics and are not direct head-to-head comparisons. The reported metric differs among systems depending on calibration method, measurement geometry, readout method, and study design.

The in vivo dose differences were 3.7 ± 1.4% (95% CI, 0.1–7.3%) for the first patient and 5.9 ± 2.1% (95% CI, 0.8–11.1%) for the second patient compared to the TPS calculations. These discrepancies include contributions from intrinsic dose uncertainty (Section 3.2.3) and extrinsic factors related to the in vivo setup. Because the dose uncertainty was < 3% for absorbed doses exceeding 1 Gy, intrinsic dosimeter uncertainty may partly account for the observed measured–calculated dose differences.

In all cases, the measured values were higher than the TPS-calculated doses. Since most measurements were taken in dose-gradient regions, localization uncertainty caused by intra-fraction anatomical motion or dosimeter positioning may have contributed to dose deviations. However, quantitative MRI-marker localization accuracy was not independently measured, and a formal sensitivity analysis of mm-level positional errors was not performed. Accordingly, the relative contribution of localization uncertainty to the measured–calculated dose differences could not be isolated from the present data. The material properties of the active layer may also have contributed to this discrepancy. Because the MRIdian KMC algorithm calculates dose to water [25], it may not fully reflect the response of the bromine-containing active layer, which includes 4% tetrabromomethane (CBr₄). The relatively high atomic number of bromine could enhance absorption of low-energy scattered photons through photoelectric interactions, resulting in a material-dependent over-response. Thus, the higher measured doses may have resulted from both localization uncertainty and the material-dependent response of the active layer. Future studies should quantify this effect using material-specific correction factors or TPS calculations with material and density overrides.

A major limitation of this study is the limited in vivo dataset, which included only two prostate cancer patients with three evaluated fractions per patient. Consequently, the present clinical data are insufficient to establish the clinical effectiveness or generalizable clinical feasibility of the proposed rectal dosimeter, and formal statistical analysis of inter-patient variability, interfractional reproducibility, or associations with clinical outcomes was not possible. The wide confidence intervals of the mean dose differences also reflect the limited precision of this preliminary dataset. Therefore, the in vivo results should be interpreted as proof-of-concept feasibility data, and further validation in a larger patient cohort with more treatment fractions is required to confirm the reproducibility, statistical robustness, and clinical utility of this rectal dosimetry system for MR-IGRT.

Another limitation of this study is that the dose comparison was based on average doses within an ROI. As polyurethane-based dosimeters allow for 3D dose analysis, follow-up research on 3D dose distributions is required. Although polyurethane-based dosimeters have the potential for 3D dose analysis, three-dimensional dose distributions were not evaluated in this study. Furthermore, achieving consistent positional reproducibility for every fraction was challenging, as shown by the sharp plan dose increases in certain fractions in Table 3. The marked interfractional variation in the TPS-calculated rectal ROI dose further emphasizes the need to improve dosimeter fixation and to quantify localization uncertainty, particularly in dose-gradient regions. Therefore, future work should also focus on improving dosimeter fixation and positioning reproducibility, particularly in dose-gradient regions.

## 5. Conclusions

We fabricated an in vivo rectal dosimeter based on a polyurethane radiochromic material with an MRI marker and a length-adjustable applicator for MR-IGRT. In this proof-of-concept clinical evaluation, in vivo measurements in two prostate cancer patients supported the preliminary feasibility of rectal in vivo dose verification by comparison with TPS-calculated doses. Further studies with larger patient cohorts and more treatment fractions are required to validate its reproducibility, statistical robustness, and clinical utility. Further refinement is also needed to improve positioning reproducibility, particularly in dose-gradient regions.

## Supporting information

S1 Dataset Underlying data for Figs 4 and 5. Spreadsheet containing the absorbed dose, net optical density, and dose uncertainty data used to generate Figs 4 and 5.
(XLSX)
